# SimHOEPI: A resampling simulator for generating single nucleotide polymorphism data with a high‐order epistasis model

**DOI:** 10.1002/qub2.42

**Published:** 2024-04-16

**Authors:** Yahan Li, Xinrui Cai, Junliang Shang, Yuanyuan Zhang, Jin‐Xing Liu

**Affiliations:** ^1^ School of Computer Science Qufu Normal University Rizhao China; ^2^ School of Information and Control Engineering Qingdao University of Technology Qingdao China; ^3^ School of Health and Life Sciences University of Health and Rehabilitation Sciences Qingdao China

**Keywords:** high‐order epistasis model, penetrance table, resampling strategy, simulation, single nucleotide polymorphisms

## Abstract

Epistasis is a ubiquitous phenomenon in genetics, and is considered to be one of main factors in current efforts to unveil missing heritability of complex diseases. Simulation data is crucial for evaluating epistasis detection tools in genome‐wide association studies (GWAS). Existing simulators normally suffer from two limitations: absence of support for high‐order epistasis models containing multiple single nucleotide polymorphisms (SNPs), and inability to generate simulation SNP data independently. In this study, we proposed a simulator SimHOEPI, which is capable of calculating penetrance tables of high‐order epistasis models depending on either prevalence or heritability, and uses a resampling strategy to generate simulation data independently. Highlights of SimHOEPI are the preservation of realistic minor allele frequencies in sampling data, the accurate calculation and embedding of high‐order epistasis models, and acceptable simulation time. A series of experiments were carried out to verify these properties from different aspects. Experimental results show that SimHOEPI can generate simulation SNP data independently with high‐order epistasis models, implying that it might be an alternative simulator for GWAS.

## 1 INTRODUCTION

With the completion of the Human Genome Project and the rapid development of high‐throughput sequencing technologies, tremendous amounts of genetic data, such as single nucleotide polymorphism (SNP) data, have been generated. Genome‐wide association studies (GWAS) have emerged as powerful for discovering genetic markers associated with complex diseases, and they often use SNPs as genetic markers.

It has been widely accepted that epistasis affects complex diseases through nonlinear interactions between SNPs [1, 2]. To analyze epistatic interactions, many epistasis detection methods have been proposed. Although evaluation of these methods with real SNP data is essential, the underlying causal SNPs or epistatic interactions are usually unknown. Therefore, it is difficult to use real SNP data to evaluate epistasis detection methods [3]. To better evaluate the performance of epistasis detection methods and enable them to identify epistatic interactions accurately and effectively in terms of real SNP data, experiments using simulation data are necessary.

Many epistasis simulators have been developed so far. For instance, GWAS simulator [4] can generate simulation data for case‐control or population samples with the sliding window algorithm. HAPGEN2 [5] has made improvements on the previous HAPGEN [6], on the one hand retaining the advantages of HAPGEN for generating realistic linkage disequilibrium (LD) patterns, and on the other hand increasing the types of models. GAMETES [7] is an epistasis simulator that uses a random architecture to generate penetrance tables of pure or strict epistasis models with specified prevalence or heritability. EpiSIM [8] can simulate SNP data with LD patterns and haplotype blocks using the Markov Chain strategy, and can calculate penetrance tables depending on either prevalence or heritability. EpiGEN [9] supports high‐order epistasis models, and generates both categorical and quantitative phenotypes. However, it generates simulation data by calling upon HAPGEN2 to produce LD patterns. Toxo [10] specifies one of either heritability or prevalence, and then maximizes the other to obtain the penetrance table. EpiReSIM [11] is a resampling simulation method for generating epistasis models without marginal effects. None of these methods is perfect in all scenarios and each has its own merits and limitations. In general, most of them only generate low‐order epistasis models and depend on other tools to generate SNP data.

In this study, we proposed a simulator, SimHOEPI, for generating high‐order epistasis models and simulating SNP data independently. Specifically, SimHOEPI calculates penetrance tables of high‐order epistasis models depending on either prevalence or heritability when given the baseline penetrance. SimHOEPI uses a resampling strategy to simulate SNP data, which needs a real SNP data set as the sampling set. Specifically, SimHOEPI generates simulation samples by resampling genotype fragments sequentially from different samples in the sampling set, and concatenating them. Highlights of SimHOEPI are the preservation of realistic minor allele frequencies (MAFs) of sampling data, the accurate calculation and embedding of high‐order epistasis models, and the acceptable simulation time. In addition, a graphical user interface (GUI) is provided for the convenience of users for calculation of epistasis models, especially for high‐order epistasis models. Hence, SimHOEPI might be an alternative simulator to GWAS.

## 2 RESULTS

Experiments were carried out to verify three SimHOEPI highlights from different aspects. First, we compared MAFs between the simulation data set and the real sampling data set to test the ability of SimHOEPI in preserving MAFs of sampling data. Second, two epistasis models were calculated and embedded in the simulation data set, and several epistasis detection methods were applied to the simulation data set to identify these two models, proving its ability in accurate calculation and embedding of high‐order epistasis models. Third, running times of SimHOEPI generating different scales of simulation data sets were recorded to verify its controllable and acceptable simulation time. Fourth, SimHOEPI was compared with some state‐of‐the‐art SNP simulators to prove its flexibility and practicality. In addition, a simple example was provided to illustrate how to calculate an epistasis model and then generate the simulation SNP data set. All above experiments were performed on a Linux machine with Intel(R) Xeon(R) Gold 5218R CPU @ 2.1 GHz and 96 GB RAM.

### 2.1 Preservation of realistic MAFs of sampling data

**FIGURE 1:**
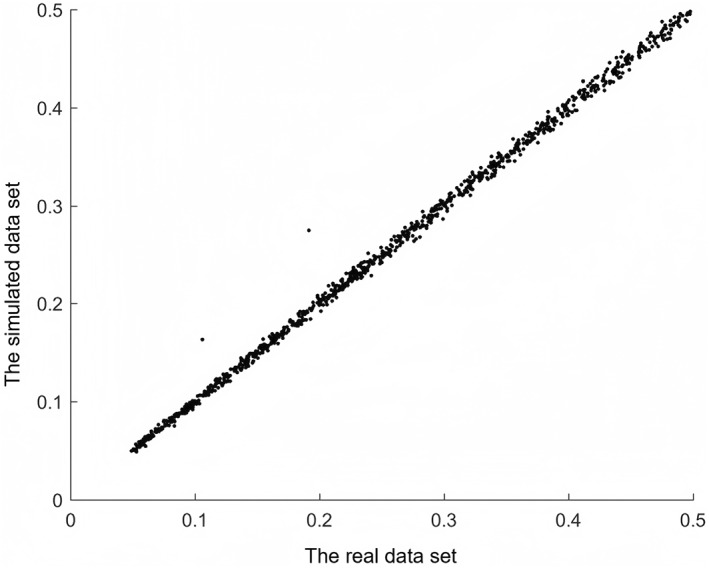
Minor allele frequencie (MAF) comparisons between the simulation and the sampling data sets.

To analyze the ability of SimHOEPI to preserve realistic MAFs of sampling data, we used it to generate a data set with 1000 SNPs and 1000 samples (500 cases and 500 controls), and then to calculate their MAFs in simulation and sampling data sets, respectively. Figure 1 shows the comparisons of MAFs between the simulation and the sampling data sets in an ascending order, where the *x*‐axis is MAFs of the sampling data, the *y*‐axis is MAFs of the simulation data, so that each point is the MAF of the same SNP locus corresponding to both the simulation data and the sampling data. Ideally, the MAFs resulting from simulation and sampling data are equal, and hence all points in the figure are on a single line. It is seen that MAFs of the simulation data generated by SimHOEPI have highly linear correlation with the MAFs of the sampling data, the Pearson’s correlation coefficient for which is 0.9991, implying that SimHOEPI can preserve the MAFs of sampling data.

### 2.2 Accurate calculation and embedding of epistasis models

To verify the accuracy of the epistasis model embedded in the simulation data generated by SimHOEPI, we adopt seven epistasis detection methods to identify epistasis models embedded in the simulation data set. First, SimHOEPI is used to generate two data sets (dataset A and dataset B). Each dataset has 4000 samples (2000 cases and 2000 controls) and 1000 SNPs. In dataset A, a 2‐order epistasis model with the SNPs (184, 660) is embedded (penetrance table is shown in Table 1), and in dataset B, a 3‐order epistasis model with the SNPs (336, 640, 8) is embedded (penetrance table is shown in Table 2).

**TABLE 1:** The penetrance table of the two‐order epistasis model.

Genotype	*AA*	*Aa*	*aa*
*BB*	0.084	0.084	0.084
*Bb*	0.084	0.210	0.210
*bb*	0.084	0.210	0.210

**TABLE 2:** The penetrance table of the three‐order epistasis model.

Genotype	*AABB*	*AABb*	*AAbb*	*AaBB*	*AaBb*	*Aabb*	*aaBB*	*aaBb*	*aabb*
*CC*	0.086	0.130	0.195	0.130	0.195	0.294	0.195	0.294	0.442
*Cc*	0.130	0.195	0.294	0.195	0.294	0.442	0.294	0.442	0.665
*cc*	0.195	0.294	0.442	0.294	0.442	0.665	0.442	0.665	1.000

Seven epistasis detection methods are then adopted to identify these two epistasis models in dataset A and B: BOOST [12], epiACO [13], IACO [14], FDHE‐IW [15], EACO [16], MACOED [17], and SIPSO [18], respectively. In the experiment, the parameters of these epistasis detection methods are set to their default values. The experimental results are shown in Table 3. It is seen that, for the 2‐order epistasis model in dataset A, all seven methods identify the epistasis model and find the true SNPs; for the 3‐order epistasis model in dataset B, epiACO, IACO, FDHE‐IW and EACO identify the model and find the true SNPs, whereas BOOST, MACOED and SIPSO cannot identify it since they are only developed for detecting 2‐order epistasis models.

**TABLE 3:** Results of seven epistasis detection methods on the simulation data.

Epistasis detection methods	Results	
	2‐order	3‐order
BOOST	**184, 660**	/
epiACO	**184, 660**	**336, 640, 708**
	184, 549	336, 640, 976
	94, 184	336, 708, 976
IACO	**184, 660**	**336, 640, 708**
	184, 658	336, 640, 905
FDHE‐IW	**184, 660**	**336, 640, 708**
	184, 730	336, 640, 856
	660, 785	336,708, 856
EACO	**184, 660**	**336, 640, 708**
	184, 494	336, 708, 747
	1, 184	336, 640, 747
MACOED	**184, 660**	/
	184, 676	/
	184, 354	/
SIPSO	**184, 660**	/
	730, 660	/
	660, 347	/

*Note*: The bold values are groundtruth SNPs in the simulated epistasis models.

### 2.3 Acceptable simulation time

**FIGURE 2:**
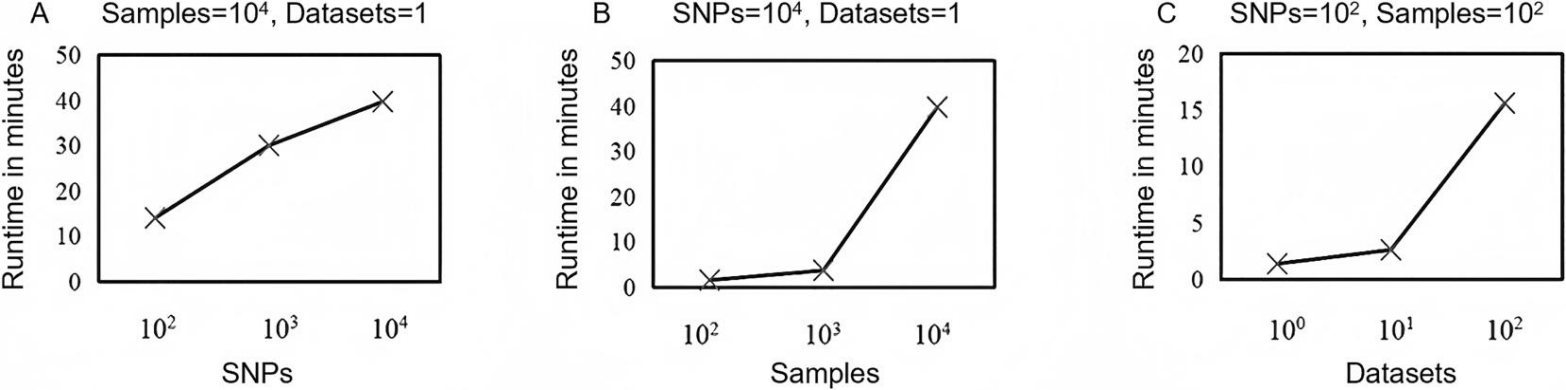
Running times of SimHOEPI under different settings.

To demonstrate that SimHOEPI can generate the simulation data within an acceptable running time, we first set different numbers of SNPs (#SNPs), samples (#samples) and data sets (#datasets) in SimHOEPI to generate simulation data sets and record their corresponding running times. To avoid outliers, the simulation process of each scene is repeated 50 times. Figure 2 shows running times of SimHOEPI under different settings, illustrating that SimHOEPI is capable of simulating large‐scale data with 10^4^ SNPs, 10^4^ samples, and 10^2^ data sets in an acceptable running time. Moreover, the running time grows almost linearly with the number of samples, SNPs, and data sets. We then set different types of epistasis models, numbers of SNPs, and orders of epistasis models to generate simulation data sets and record their corresponding running times. Table 4 presents running times for generating different simulation data sets. By analyzing these results, it can be seen that when SimHOEPI embeds models with the same order to generate data sets with the same number of SNPs, running times are very close. Therefore, SimHOEPI is not sensitive to the model type, and the main time requirement in generating the simulation data is to read the sampling data set because the data are usually large.

**TABLE 4:** Running times with different models and single nucleotide polymorphism (SNP) numbers.

Model	Order	10,000 SNPs		50,000 SNPs	
		*h* ^2^	Time (min)	*h* ^2^	Time (min)
Additive	2	0.051	4.56	0.054	11.16
Multiplicative	2	0.103	4.90	0.048	10.57
Threshold	2	0.211	4.76	0.210	10.52
Additive	3	0.031	6.72	0.029	16.00
Multiplicative	3	0.017	7.84	0.205	16.22
Threshold	3	0.177	6.58	0.205	14.59

### 2.4 Comparison with existing simulators

We select three representative simulators for comparison with SimHOEPI, namely, epiSIM [8], GAMETES [7], and Toxo [10], all of which are capable of computing penetrance tables. Comparison results are shown in Table 5. Compared with other simulators, SimHOEPI has several advantages. First, it can well preserve MAFs of the sampling data, and can specify MAFs matching SNPs in the model. SimHOEPI not only provides convenience to users but also makes the simulation data fit the sampling data more closely. Second, SimHOEPI can calculate high‐order epistasis models and provide a graphical interface that allows users to focus more on the design of epistasis models rather than simulation techniques and user’s manual.

**TABLE 5:** Comparison results of SimHOEPI with existing simulators.

	epiSIM	GAMETES	Toxo	SimHOEPI
Generate realistic MAFs	×	×	×	√
Support MAF specification	√	√	√	√
Simulate high‐order epistasis models	×	√	√	√
Not depend on other software	√	√	×	√
Graphical user interface	√	√	×	√

### 2.5 Usage example

**FIGURE 3:**
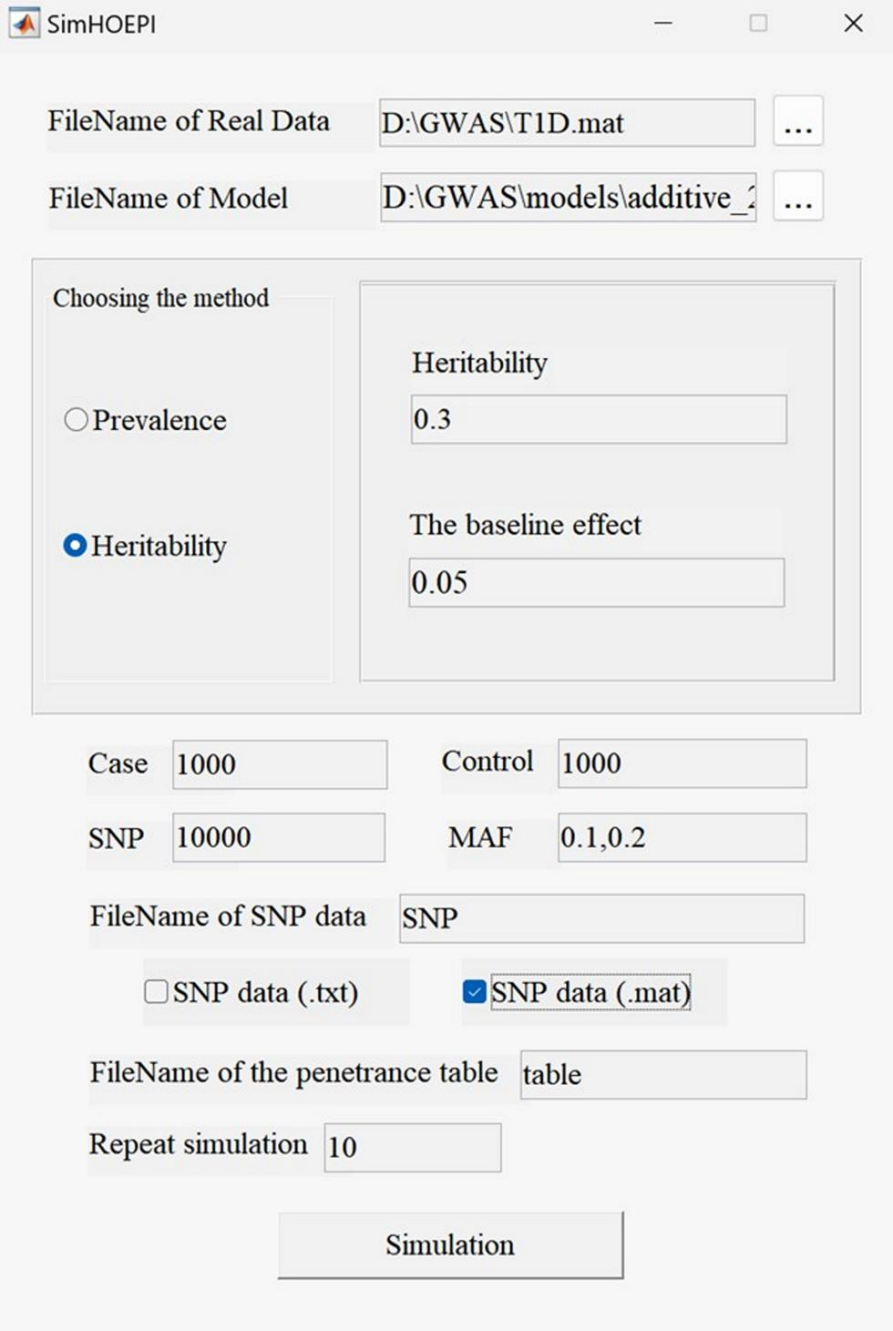
The example interface of SimHOEPI.

SimHOEPI provides a user‐friendly GUI. Using SimHOEPI to generate simulation data is mainly divided into four steps: inputting the required files, selecting the calculation method, setting general parameters, and generating simulation data. First, SimHOEPI requires two input files: the sampling data set file and the model file. The sampling data set in SimHOEPI is represented as a matrix, where each row represents a sample and each column represents a SNP. The model file is written in comma separated values format, where the rows are the different genotypes and two columns are the penetrance function expressions corresponding to the combination genotypes. There are two variables in the expression function, *x* and *y*, which correspond to the baseline penetrance *α* and the relative penetrance *f*, respectively. Second, SimHOEPI provides two ways to calculate epistasis models. The users only need to click buttons in the GUI to select the calculation method and input corresponding parameters to calculate the epistasis model. Third, users can freely set and select several general parameters, including cases, controls, SNPs, MAFs, the output forms of simulation data, etc, default parameter values of which are also given in the original interface for reference. Finally, after clicking the simulation button, SimHOEPI calculates the epistasis model and generates the simulation data set. SimHOEPI then outputs data set files and the final model files. As shown in Figure 3, we give an interface for an example of using SimHOEPI to generate simulation data.

## 3 DISCUSSION

The main contribution of this work is to develop a simulator, SimHOEPI, which is capable of calculating penetrance tables of high‐order epistasis models and of using a resampling strategy to generate simulation data. SimHOEPI allows selection of prevalence or heritability to calculate the epistasis model with the baseline penetrance provided by users. Moreover, for convenience, SimHOEPI provides a GUI. Highlights of SimHOEPI are the preservation of realistic MAFs of sampling data, the accurate calculation and embedding of high‐order epistasis models, and the acceptable simulation time. In addition, a series of experiments were carried out. Experimental results show that SimHOEPI can generate simulation SNP data independently with high‐order epistasis models, implying that it might be an alternative simulator for GWAS.

SimHOEPI also has several limitations. First, SimHOEPI can only calculate penetrance tables of epistasis models displaying marginal effects (eME models) and does not support the calculation of penetrance tables of epistasis models displaying no marginal effects (eNME models). Second, the current version of SimHOEPI does not consider interactions between environmental and genetic factors. These two limitations inspire us to continue this work.

## 4 METHOD

### 4.1 Resampling for generating samples

To generate simulation data, SimHOEPI first requires a sampling dataset. Here we use a SNP dataset of Type 1 Diabetes (T1D) as an example. The T1D real SNP dataset is collected from the Wellcome Trust Case Control Consortium, which includes 377,047 SNPs and 5004 samples (3004 cases and 1999 controls) [19]. The T1D real SNP dataset is processed as a matrix, where each row represents a sample and each column represents a SNP. The elements in the matrix are the genotypes *g*_*i*_, where 1 represents homozygous common genotype (*AA*), 2 represents heterozygous genotype (*Aa*) and 3 represents homozygous minor genotype (*aa*). These samples are divided into controls and cases, where the former is labeled by 0 and the latter is labeled by 1.

The generation process of these samples is described as follows. First, SimHOEPI selects a group of SNPs at random locations in the sampling dataset to generate samples of simulation data based on a user‐specified number of SNPs. Second, samples in the sampling dataset labeled as control are selected for simulating epistasis models. Third, these sampling data processed above are used to generate SNP simulation data by a resampling strategy. To generate a sample of simulation data, each sample of these preprocessed sampling data is randomly segmented according to the column, with each segment having a random number of SNPs. Therefore, these preprocessed sampling data are divided into many groups where each group has SNP fragments with the same locus. A sample of simulation data is generated by randomly selecting a segment in each group and splicing these segments in turn. For example, if the sampling dataset has 200 samples (rows), and each sample is divided into 50 segments. The simulation data for a sample is generated by randomly selecting a segment from the first group, the second group, the third group, and so on, until all 50 groups have been selected. Then the 50 selected segments are spliced together in turn to generate a sample of simulation data. Thus, the simulation data retains the basic patterns of LD and MAFs as observed in the sampling data set [8, 20, 21].

### 4.2 Calculating the epistasis models

It is the penetrance function, also described as the penetrance table that bridges the relationship between genotype and disease risk. Penetrance value is the probability of being affected while given the genotype *g*_*i*_ of a sample, which is usually denoted as *P*(*D*|*g*_*i*_). In sufficiently large populations, SNPs under not selective pressure and random mating typically result in genotype frequencies satisfying the Hardy‐Weinberg Law [22]. Like most SNP simulators, SimHOEPI adopts the assumption of Hardy‐Weinberg Equilibrium so that given the MAF of SNP *k* in the epistasis model as MAF_*k*_ where *k* ∈{1, 2, …, *K*}, the genotype frequencies of SNP *k* corresponding to (*AA, Aa, aa*) can be determined as

(1) $$q_{k}={\left(\left(1-\text{MA}F_{k}\right)\right.}^{2},2\text{MA}F_{k}\left(1-\text{MA}F_{k}\right),\text{MA}{F_{k}}^{2}).$$

Assuming linkage equilibrium among *K* SNPs, the frequency of the combination‐genotype *P*(*g*_*i*_) is constant for fixed MAF_*k*_, being the product of frequencies of the corresponding genotype of each SNP.

The eME model is a subtype of epistasis models, in which one or more SNPs have marginal effects and the epistasis effect. The effect of the eME model has been divided into their main effect and epistatic effect [23]. For the convenience of calculation, the penetrance of eME model is expressed as a function composed of the baseline penetrance *α* and the relative penetrance *f* [8, 24, 25], which can be written as,

(2) $$P\left(D|g_{i}\right)=F\left(\alpha ,f\right).$$

There are two parameters to model the characteristics of the population, prevalence (*P*(*D*)) and heritability (*h* ^2^). Prevalence is the probability that a sample will be affected by an epistasis model and it is the sum of the product of the genotype frequencies and the corresponding penetrance values of combination genotypes in an epistasis model. The prevalence of a *K*‐order model can be calculated by

(3) $$P\left(D\right)=\sum_{i}^{3^{K}}P\left(D|g_{i}\right)P\left(g_{i}\right).$$

Heritability represents the amount of phenotypic variation that corresponds to genetic variation [10]. Phenotypes may be caused by genetic factors, environmental factors, or the interaction of genes and environments. Therefore, the range of heritability is between 0 and 1. The heritability of a *K*‐order model is

(4) $$h^{2}=\frac{\sum_{i}^{3^{K}}{\left(P\left(D|g_{i}\right)-P\left(D\right)\right)}^{2}P\left(g_{i}\right)}{P\left(D\right)\left(1-P\left(D\right)\right)}.$$

The calculation of epistasis model is to determine 3^*K*^ penetrance values where a penetrance value corresponds to a combination genotype. For an eME model, the calculation of the model is to determine the baseline penetrance *α* and the relative penetrance *f*. When calculating a *K*‐order eME model, SimHOEPI first needs to select the *K* loci in this eME model based on MAFs specified by the user. SimHOEPI determines the search range in steps of 0.01 according to these MAFs, within the range of −0.01 to +0.01. Among SNPs satisfying the above conditions, *K* SNPs are randomly selected as the loci for the eME model in the simulation data. If the number of searched SNPs is less than *K*, the search range is dynamically expanded. Second, the frequency of the combination genotype *P*(*g*_*i*_) is calculated using the MAFs of these selected SNPs. SimHOEPI calculates the relative penetrance value *f* using one of the two parameters (prevalence or heritability) and baseline penetrance value *α*. Given *h* ^2^ or *P*(*D*), MAF_*k*_ and *α*, the solution of *f* can be easily obtained, meaning that an eME model is obtained. If the calculated penetrance of the eME model has a value greater than 1, then all the penetrance values in the table are transformed by the function *S* to ensure that penetrance values are between 0 and 1. The calculation process of *S* is,

(5) $$S=\frac{P{\left(D|g_{i}\right)}^{\prime }}{1+M}$$

where $P{\left(D|g_{i}\right)}^{\prime }$ is the penetrance value that needs to be transformed, and *M* is the maximum value in the penetrance table. After transforming these penetrance values, the final epistasis model is obtained.

### 4.3 Generating the labels of samples

To embed the epistasis model into a simulation data set, SimHOEPI generates a label for each sample and divides these samples into case group and control group. The label of a sample is jointly determined according to the calculated penetrance values and the combination genotypes at the loci of the epistasis model. The probability that a sample is labeled as a case is the penetrance value *P*(*D*|*g*_*i*_) in the epistasis model if the model affects it. In the final outputs, SimHOEPI can output sample‐balanced data and sample‐unbalanced data.

### 4.4 Implementation

**FIGURE 4:**
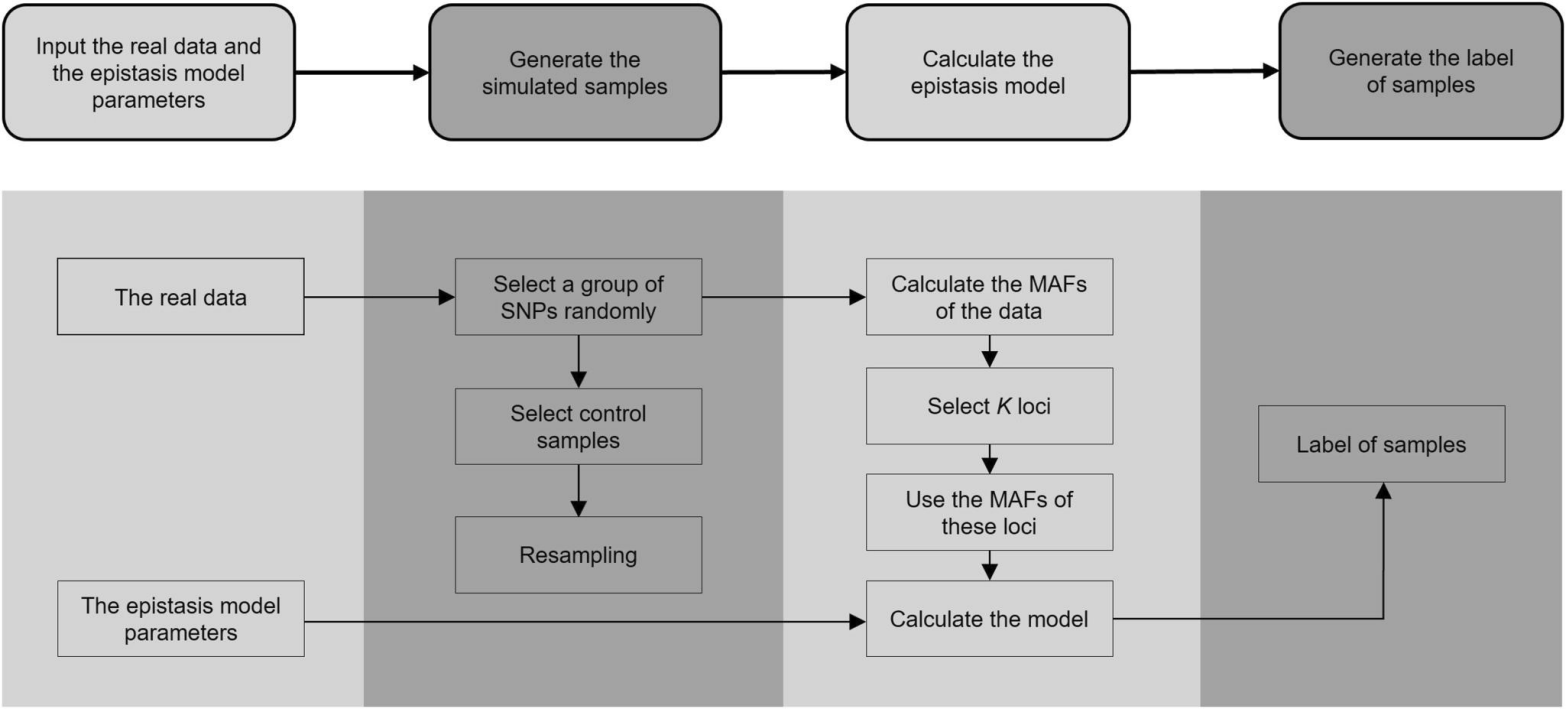
Flow chart of SimHOEPI.

The current version of SimHOEPI is a MATLAB program, which has three stages: generating samples, calculating epistasis models, and generating the labels of samples. The flow chart that gives an overview of SimHOEPI is shown in Figure 4. The input files for the program are sampling SNP dataset files and epistasis model files. The SNP data is a matrix where each row represents a sample and each column represents a SNP. The epistasis model file is a penetrance table and the information in the table consists of genotypes and their corresponding penetrance value. The user determines the order of the epistasis model in the simulation data according to the input model file. SimHOEPI uses the sampling data to generate simulation data by a resampling strategy. The output files are SNP simulation data and an epistasis model file. The program allows users to specify an array of parameters such as the number of SNPs, the number of samples, the MAFs and the number of simulation data sets.

## AUTHOR CONTRIBUTIONS

**Yahan Li** and **Xinrui Cai**: Conceptualization. **Yahan Li** and **Junliang Shang**: Methodology. **Yuanyuan Zhang** and **Jin‐Xing Liu**: Validation. **Junliang Shang** and **Yuanyuan Zhang**: Resources. **Yahan Li**: Data curation. **Yahan Li**: Writing – original draft preparation. **Xinrui Cai** and **Junliang Shang**: Writing – review and editing. **Yahan Li** and **Yuanyuan Zhang**: Visualization. **Jin‐Xing Liu** and **Xinrui Cai**: Supervision. **Junliang Shang**: Project administration. **Junliang Shang** and **Jin‐Xing Liu**: Funding acquisition. All authors have read and agreed to the published version of the manuscript.

## CONFLICT OF INTEREST STATEMENT

The authors Yahan Li, Xinrui Cai, Junliang Shang, Yuanyuan Zhang and Jin‐Xing Liu declare that they have no conflict of interest or financial conflicts to disclose.

## ETHICS STATEMENT

This article does not contain any studies with human or animal subjects performed by any of the authors.
